# Disparities in Lung-Protective Ventilation in the United States

**DOI:** 10.7759/cureus.29834

**Published:** 2022-10-02

**Authors:** Michelle L Malnoske, Caroline M Quill, Amelia K Barwise, Anthony P Pietropaoli

**Affiliations:** 1 Division of Pulmonary and Critical Care Medicine, University of Rochester Medical Center, Rochester, USA; 2 Division of Pulmonary and Critical Care Medicine, Mayo Clinic, Rochester, USA

**Keywords:** critical care, sex, insurance coverage, lung injury, respiratory failure, tidal volume, mechanical ventilation

## Abstract

Background

The objective of our study was to determine whether disparities exist in the use of lung-protective ventilation for critically ill mechanically ventilated patients in the United States based on gender, race/ethnicity, or insurance status.

Methods

This was a secondary data analysis of a prospective multicenter cohort study conducted from 2010 to 2012. The outcome of interest was the proportion of patients receiving tidal volume > 8 mL/kg predicted body weight (PBW).

Results

There were 1,595 patients in our primary analysis (710 women, 885 men). Women were more likely to receive tidal volumes > 8 mL/kg PBW than men (odds ratio [OR] = 3.42, 95% confidence interval [CI] = 2.67-4.40), a finding largely but not completely explained by gender differences in height. The underinsured were significantly more likely to receive tidal volume > 8 mL/kg PBW than the insured in multivariable analysis (OR = 1.54, 95% CI = 1.16-2.04). The prescription of > 8 mL/kg PBW tidal volume did not differ by racial or ethnic categories.

Conclusions

In this prospective nationwide cohort of critically ill mechanically ventilated patients, women and the underinsured were less likely than their comparators to receive lung-protective ventilation, with no apparent differences based on race/ethnicity alone.

## Introduction

Disparities exist in healthcare delivery and clinical outcomes among critically ill patients based on gender, race, and insurance status [1-7].

Lung-protective ventilation is often used for patients with acute respiratory distress syndrome (ARDS) and also for patients without ARDS [8], with several studies indicating lower risk of lung injury and other adverse outcomes in non-ARDS patients [9-11]. Few studies have specifically investigated whether tidal volumes differ based on gender, race, and insurance status among unselected critically ill mechanically ventilated patients [12].

The goal of this study was to explore whether gender, race, and insurance status influenced the use of lung-protective ventilation. To accomplish this goal, we conducted a secondary analysis of The United States Critical Illness and Injury Trials Group-Critical Illness Outcomes Study (USCIITG-CIOS), a multicenter, prospective cohort study designed to evaluate the impact of ICU protocols on patient outcomes [13,14]. We hypothesized that potentially injurious tidal volumes would be differentially applied based on gender, race/ethnicity, and insurance status. Preliminary analyses from this study were previously presented at the American Thoracic Society International Conference on May 23, 2018, and published in abstract form [15].

This article was previously posted to the Research Square pre-print server on January 5, 2022.

## Materials and methods

Study design, setting, and patients

The details of USCIITG-CIOS have been previously described [13,14]. In brief, this was a prospective cohort study of 6,179 critically ill adult patients from 59 primarily academic intensive care units (ICUs) across the United States. Participating ICUs enrolled newly admitted patients one day per week, with 5-10 days between enrollment days, between July 2010 and March 2012. Data collection elements included demographic characteristics, height, and mechanical ventilation settings abstracted from review of the electronic medical record by trained study personnel at each participating center. Mechanical ventilation parameters were collected from the respiratory flowsheets of the medical record at a single time point at approximately 8:00 am the day of data collection. Patients present in the ICU during the prior data collection day or discharged before the first data collection day were not enrolled. All participating sites received approval from their institutional review boards for data collection with a waiver of informed consent.

Exposure variables

The independent variables of interest were gender, race/ethnicity, and insurance status. Race was nominally categorized as White (the base category), African American, Asian-Pacific Islander, and American Indian/Alaskan Native. Ethnicity was binarily categorized as not Hispanic or Latino or Hispanic or Latino. Underinsured patients were those with Medicaid-only coverage, self-pay, or unknown insurance, and insured patients were those with any Medicare or commercial/private insurance [2]. We performed a sensitivity analysis that excluded Medicare patients from the analysis of insurance status to assess the likelihood of confounding by age and comorbid conditions [4].

Outcome variable

The outcome variable was prescription of a tidal volume/predicted body weight (VT/PBW) > 8 mL/kg. We chose this outcome because it is a potentially harmful threshold used in prior studies of ventilatory practices [12,16,17] and could be differentially applied in patients based on gender, race/ethnicity, or insurance status. PBW was calculated using the formulas employed by the ARDSnet investigators [18].

Statistical analysis

Our primary analysis was a complete case analysis that included patients with non-missing values for race, ethnicity, tidal volume, and height. We also performed a secondary analysis that included patients with missing values for these variables using multiple imputation. The details of the multiple imputation methods are presented in the supplementary methods and Supplementary Tables 5-7.

Descriptive statistics were performed for all dependent and independent variables of interest. Continuous variables with a normal and skewed distribution are reported as mean ± standard deviation or median [interquartile range], respectively. Categorical variables are expressed as proportions. Relationships between dichotomous variables were examined using the chi-square test, and relationships between continuous variables were analyzed using the Kruskal-Wallis test. We used clinical judgment and prior literature to construct directed acyclic graphs conceptualizing covariables that might confound or mediate relationships between the independent and dependent variables of interest [19-21]. These covariables were included together with the predictor variable of interest in multivariable logistic regression models. The outcome variable was VT/PBW > 8 mL/kg. The “cluster” option in Stata was used for estimation of the variance-covariance matrix in all logistic models. This option relaxes the assumption of independent observations within groups, adjusting the standard errors and confidence intervals (CIs) to account for the possibility that care of patients within individual ICUs was correlated [22].

Mediation analysis was conducted according to the methods of Pearl [23] to probe relative contributions of gender and height on tidal volume > 8 mL/kg PBW. Statistical analyses were conducted with Stata version 14.2 (2015, Stata Statistical Software, StataCorp LP, College Station, TX).

## Results

Patient characteristics

**Figure 1:**
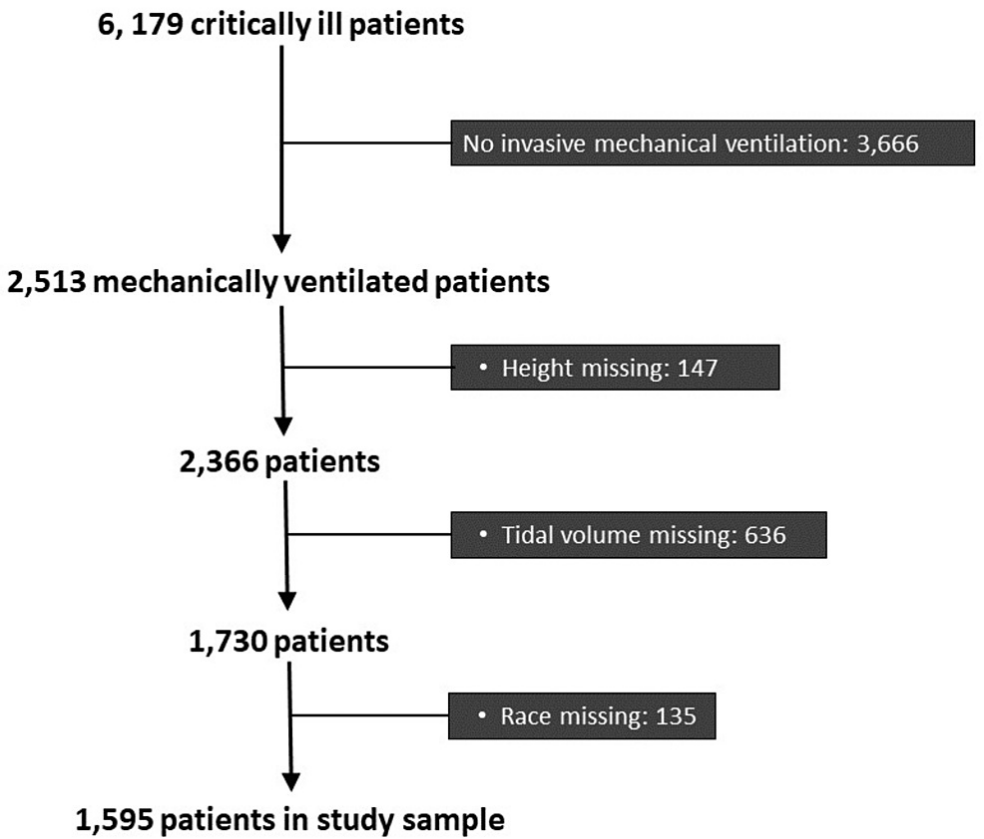
Derivation of the study sample.

We enrolled 6,179 critically ill patients from 59 ICUs, of which 2,513 patients received mechanical ventilation. Race was missing in 193 patients, tidal volume in 689 patients, and height in 147 patients. After exclusion of patients with one or more of these missing variables, 1,595 patients remained for the complete case analysis (Figure 1).

The characteristics of mechanically ventilated patients in the complete case analysis are shown in Tables 1, 2.

**Table 1:** Patient characteristics by sex and insurance status (complete case analysis) *Actual body weight was missing in 11 patients.**Infection types were as follows: pulmonary = 215 (46%), urinary = 47 (10%), abdominal = 45 (9%), central nervous system = 9 (2%), skin/soft tissue = 33 (7%), bloodstream = 73 (15%), other = 19 (4%), unknown = 31 (7%). †Mortality status, ICU length of stay (LOS), and hospital LOS were missing in 114 patients. Abbreviations: PBW, predicted body weight; BMI, body mass index; APACHE, acute physiology and chronic health evaluation; SOFA, sequential organ failure assessment; LOS, length of stay; COPD, chronic obstructive pulmonary disease; HIV, human immunodeficiency virus; AIDS, acquired immunodeficiency syndrome

		Gender			Insurance status		
Variable	Total (n = 1,595)	Women (n = 710)	Men (n = 885)	p-Value	Underinsured (n = 338)	Insured (n = 1,257)	p-Value
Age (years)	61 (51 – 71)	62 (52 – 73)	60 (50 – 70)	0.001	52 (41 – 59)	64 (54 – 74)	<0.001
Height (cm)	170 (162 – 178)	162 (157 – 167)	177 (170 – 182)	<0.001	170 (162 – 178)	170 (160 – 178)	0.45
PBW (kg)	64 (54 – 73)	54 (50 – 59)	72 (66 – 77)	<0.001	64 (55 – 87)	64 (54 – 92)	0.09
Weight (kg)*	81 (67 – 98)	72 (60 – 93)	85 (73 – 102)	<0.001	80 (66 – 98)	81 (67 – 98)	0.28
BMI (cm/m^2^)	28 (24 – 34)	28 (23 – 35)	27 (24 – 33)	0.10	27 (23 – 33)	28 (24 – 34)	0.07
APACHE II score	21 (16 – 26)	21 (16 – 25)	21 (16 – 26)	0.96	19 (14 – 24)	21 (16 – 26)	<0.001
SOFA score	7 (4 – 10)	6 (4 – 9)	7 (5 – 10)	<0.001	6 (4 – 10)	7 (4 – 10)	0.18
Hospital mortality^†^	437 (30%)	191 (28%)	246 (30%)	0.052	86 (30%)	351 (30%)	0.25
Hospital LOS (days)^†^	17 (10 – 30)	17 (10 – 29)	17 (10 – 31)	0.49	17 (9 – 33)	17 (10 – 30)	0.62
ICU LOS (days)^†^	10 (5-18)	10 (5 – 17)	10 (5 – 10)	0.24	10 (5 – 18)	10 (5 – 18)	0.28
Comorbid conditions							
Heart failure	271 (17%)	135 (19%)	136 (15%)	0.054	54 (16%)	217 (17%)	0.58
COPD	423 (26%)	212 (30%)	211 (24%)	0.007	67 (20%)	356 (28%)	0.002
Cancer	338 (21%)	139 (20%)	199 (22%)	0.16	43 (12%)	295 (23%)	<0.001
Chronic kidney disease	261 (16%)	125 (18%)	136 (15%)	0.23	39 (12%)	222 (18%)	0.007
Chronic liver disease	183 (11%)	72 (10%)	111 (12%)	0.14	48 (14%)	135 (11%)	0.08
HIV/AIDS	59 (4%)	23 (3%)	36 (4%)	0.38	29 (9%)	30 (2%)	<0.001
Admission diagnosis category							
Respiratory	865 (54%)	389 (55%)	476 (54%)	0.69	184 (54%)	681 (54%)	0.93
Infectious**	472 (30%)	229 (32%)	243 (27%)	0.037	99 (30%)	373 (30%)	0.89
Cardiovascular	467 (29%)	200 (28%)	267 (30%)	0.38	85 (25%)	382 (30%)	0.060
Gastrointestinal	236 (15%)	102 (14%)	134 (15%)	0.66	44 (14%)	192 (15%)	0.30
Trauma	101 (6%)	27 (4%)	74 (8%)	<0.001	34 (10%)	67 (5%)	0.002
Endocrine	101 (6%)	48 (7%)	53 (6%)	0.53	24 (7%)	77 (6%)	0.51
Other	235 (15%)	106 (15%)	129 (14%)	0.84	42 (12%)	193 (15%)	0.18
Admission source				0.30			<0.001
Emergency department	715 (45%)	322 (45%)	393 (44%)		196 (58%)	519 (41%)	
Hospital floor	315 (20%)	131 (18%)	184 (21%)		60 (18%)	255 (20%)	
Operating room	255 (16%)	105 (15%)	150 (17%)		31 (9%)	224 (18%)	
Outside hospital	252 (16%)	123 (17%)	129 (15%)		45 (13%)	207 (16%)	
Other	58 (4%)	29 (4%)	29 (3%)		6 (2%)	52 (4%)	

**Table 2:** Patient characteristics by racial and ethnic categories Note: values refer to median (interquartile range) or number (percentage). *Infection types were as follows: pulmonary = 215 (46%), urinary = 47 (10%), abdominal = 45 (9%), central nervous system = 9 (2%), skin/soft tissue = 33 (7%), bloodstream = 73 (15%), other = 19 (4%), unknown = 31 (7%). †Mortality status, ICU LOS, and hospital LOS were missing in 114 patients. Abbreviations: PBW, predicted body weight; BMI, body mass index; APACHE, acute physiology and chronic health evaluation; SOFA, sequential organ failure assessment; LOS, length of stay; COPD, chronic obstructive pulmonary disease; HIV, human immunodeficiency virus; AIDS, acquired immunodeficiency syndrome

		Race					Ethnicity		
Variable	Total n = (1,595)	White (n = 1,113)	Black (n = 424)	Asian (n = 51)	American Indian/Alaska native (n = 7)	p-value	Non-Hispanic or Latino (n = 1,544)	Hispanic or Latino (n = 51)	p-Value
Age (years)	61 (51 – 71)	62 (52 – 74)	58 (48 – 67)	65 (54 – 78)	55 (53 – 61)	<0.001	61 (51 – 72)	58 (37 – 67)	0.008
Height (cm)	170 (162 – 178)	170 (162 – 178)	170 (162 – 177)	165 (157 – 173)	162 (160 – 173)	0.004	170 (162 – 178)	165 (160 – 173)	0.036
PBW (kg)	64 (54 – 73)	64 (54 – 73)	63 (55 – 72)	60 (52 – 68)	55 (52 – 68)	0.35	64 (54 – 73)	60 (65 – 69)	0.41
Weight (kg)	81 (67 – 98)	82 (68 – 99)	80 (67 – 97)	64 (58 – 80)	78 (61 – 100)	<0.001	81 (67 – 98)	79 (68 – 94)	0.39
BMI (cm/m^2^)	28 (24 – 34)	28 (24 – 34)	27 (23 – 84)	24 (22 – 27)	28 (24 – 38)	<0.001	28 (24 – 34)	28 (25 – 32)	0.92
APACHE II score	21 (16 – 26)	21 (16 – 25)	20 (16 – 26)	21 (17 – 24)	23 (20 – 27)	0.77	21 (16 – 26)	21 (17 – 25)	0.92
SOFA score	7 (4 – 10)	7 (4 – 10)	7 (4 – 10)	7 (4 – 10)	9 (5 – 10)	0.73	7 (4 – 10)	7 (5 – 11)	0.83
Hospital mortality^†^	437 (30%)	293 (28)	120 (30)	22 (43)	2 (33)	0.16	427 (30)	10 (20)	0.16
Hospital LOS (days)^†^	17 (10 – 30)	17 (10 – 30)	18 (9 – 31)	14 (6 – 36)	16 (15 – 20)	0.84	17 (10 – 30)	16 (8 – 34)	0.59
ICU LOS (days)^†^	10 (5-18)	10 (5 – 17)	10 (5- 18)	10 (4 – 21)	12 (5 – 20)	0.99	10 (5 – 18)	8 (4 – 20)	0.41
Comorbid conditions									
Heart failure	271 (17%)	167 (15)	99 (24)	4 (8)	1 (14)	<0.001	266 (17)	5 (10)	0.16
COPD	423 (26%)	309 (28)	104 (24)	9 (18)	1 (14)	0.23	416 (27)	7 (14)	0.035
Cancer	338 (21%)	263 (23)	62 (15)	11 (22)	2 (28)	0.002	331 (21)	7 (14)	0.18
Chronic kidney disease	261 (16%)	141 (13)	113 (27)	7 (14)	0 (0)	<0.001	258 (17)	3 (6)	0.040
Chronic liver disease	183 (11%)	121 (11)	53 (12)	7 (14)	2 (28)	0.38	173 (11)	10 (20)	0.064
HIV/AIDS	59 (4%)	14 (1)	44 (10)	0 (0)	1 (14)	<0.001	58 (4)	1 (2)	0.50
Admission diagnosis category									
Infectious*	472 (30%)	291 (26)	168 (40)	12 (24)	1 (14)	<0.001	460 (30)	12 (24)	0.34
Cardiovascular	467 (29%)	300 (27)	150 (35)	16 (31)	1 (14)	0.01	462 (30)	5 (10)	0.002
Gastrointestinal	236 (15%)	170 (15)	60 (14)	5 (10)	1 (14)	0.72	231 (15)	5 (10)	0.31
Trauma	101 (6%)	73 (6)	24 (6)	3 (6)	1 (14)	0.76	98 (6)	3 (6)	0.89
Endocrine	101 (6%)	64 (6)	35 (8)	2 (4)	0 (0)	0.24	98 (6)	3 (6)	0.89
Other	235 (15%)	166 (15)	59 (14)	8 (16)	2 (28)	0.72	230 (15)	5 (10)	0.31
Admission source						<0.001			0.25
Emergency department	715 (45%)	444 (40)	246 (58)	22 (43)	3 (43)	--	685 (44)	30 (59)	--
Hospital floor	315 (20%)	215 (19)	86 (20)	11 (22)	3 (43)	--	305 (20)	10 (20)	--
Operating room	255 (16%)	201 (18)	44 (10)	9 (18)	1 (14)	--	249 (16)	6 (12)	--
Outside hospital	252 (16%)	212 (19)	33 (8)	7 (14)	0 (0)	--	248 (16)	4 (8)	--
Other	58 (4%)	41 (4)	15 (4)	2 (4)	0 (0)	--	57 (4)	1 (2)	--

A total of 26% (n = 411) of the patients in this cohort were diagnosed with ARDS, and 27% (435) of mechanically ventilated patients in this cohort received tidal volumes above 8 mL/kg PBW. There was no difference in hospital mortality in those who received lung-protective ventilation (31%) vs those who received tidal volumes above 8 mL/kg PBW (26%, p = 0.11).

Complete case analysis

Relationship Between Gender and Provision of Lung-Protective Ventilation

**Figure 2:**
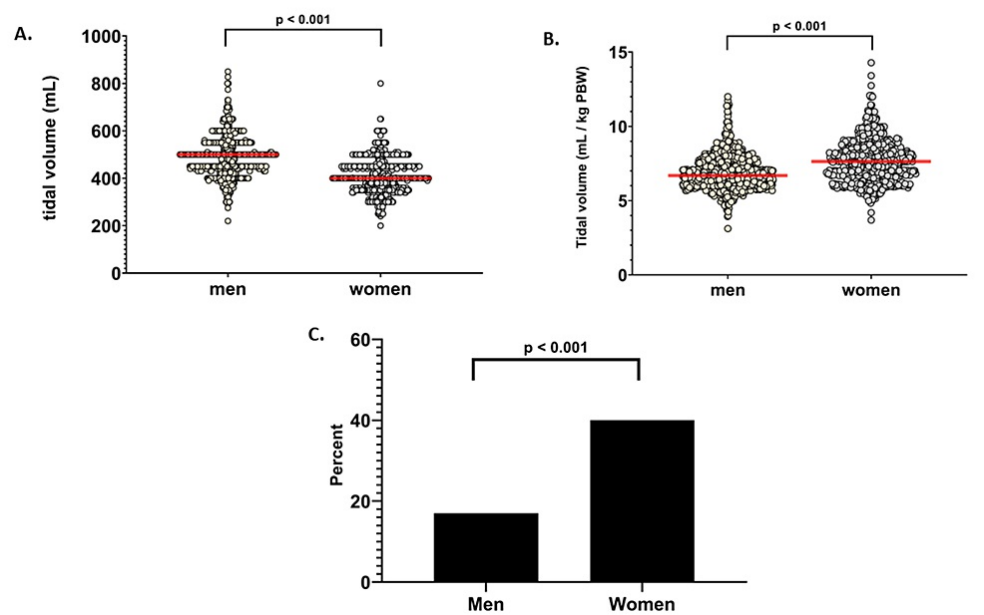
Tidal volume parameters in men vs women (A) Unadjusted tidal volume in men vs women, (B) Tidal volume adjusted for predicted body weight in men vs women. (C) Percentage of men vs women receiving tidal volume > 8 mL/kg predicted body weight. Dot plots show distributions of values, with the median value indicated by the horizontal line. Comparisons were analyzed using the rank-sum test or chi-square test.

Unadjusted tidal volumes were lower in women vs men (400 [360-450] mL vs 500 [450-550] mL, Table 3 and Figure 2).

**Table 3:** Relationships of lung-protective ventilation with gender and insurance status Note: Values refer to median (interquartile range) or number (percentage) †Adjusted for age (continuous), height (continuous), total number of comorbidities (0-5). § Adjusted for age (continuous), post-operative from elective surgery status, race, ethnicity, total number of comorbidities (0-5). Abbreviations: PBW, predicted body weight

	Gender			Insurance status		
	Women (n= 710)	Men (n = 885)	p-Value	Underinsured (n = 338)	Insured (n = 1,257)	p-Value
Tidal volume (mL)	400 (360 – 450)	500 (450 – 550)	<0.001	450 (400 – 500)	450 (400 – 500)	0.03
Tidal volume/PBW (mL/kg)	7.6 (6.7 – 8.6)	6.7 (6.0 – 7.6)	<0.001	7.1 (6.4 – 8.2)	7.0 (6.2 – 8.0)	0.09
Tidal volume > 8 mL/kg PBW	288 (40%)	147 (17%)	<0.001	105 (31%)	330 (26%)	0.08
unadjusted odds ratio	3.43 (2.67 – 4.40)	1 (ref)	<0.001	1.26 (0.92 – 1.74)	1 (ref)	0.15
Height-adjusted odds ratio	1.28 (0.91 – 1.80)	1 (ref)	0.15	---	---	--
Multivariable adjusted odds ratio (all variables)	1.28 (0.92 – 1.77)^†^	1 (ref)	0.14	1.55 (1.15 – 2.07)^§^	1 (ref)	0.003

However, women received higher tidal volume than men when adjusted for PBW (7.6 [6.7-8.6] mL/kg in women vs 6.7 [6.0-7.6] in men) and were more likely to receive tidal volumes above 8 mL/kg PBW (40% of women vs 17% of men, odds ratio [OR] = 3.43 [2.67-4.40]).

**Figure 3:**
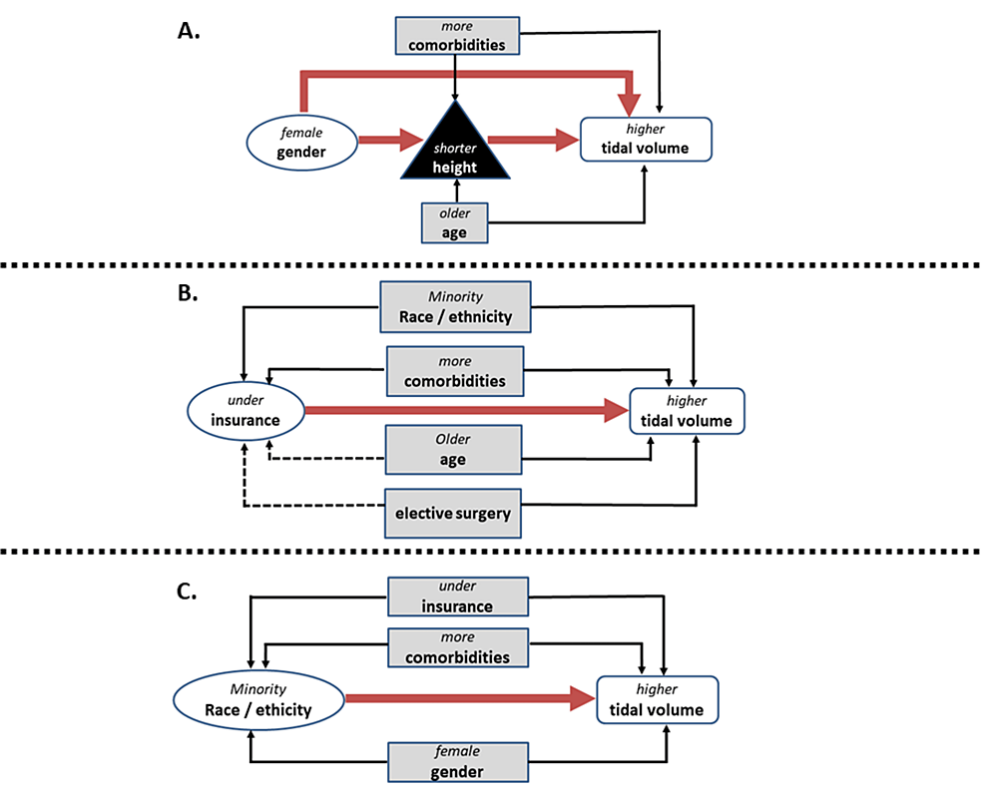
Directed acyclic graphs modeling hypothesized relationships between exposures (ovals) and outcome of interest (rectangles with white background) Proposed causal pathways are diagramed in thick solid arrows. Potential confounders are diagramed in shaded rectangles. Relationships between potential confounders and other variables are diagramed as thin arrows. Positive relationships between potential confounders and other variables are those that increase the probability of the other variable, designated by thin solid arrows. Negative relationships between potential confounders and other variables are those that decrease the probability of the other variable, designated by thin dashed arrows. Mediators are designated by black triangles. A. Theorized causal association diagram between female gender (exposure) and higher tidal volume (outcome). There are two possible causal pathways diagramed: one that includes shorter height as a mediator (the indirect path) and one goes directly from female gender to higher tidal volume (the direct path). Medical comorbidities and older age are diagramed as possible confounders of the relationship between height (mediator) and tidal volume (outcome). B. Theorized causal association diagram between underinsurance (exposure) and higher tidal volume (outcome). Minority race/ethnicity and more comorbidities are diagramed as potential confounders sharing positive associations with both the exposure and the outcome. Older age and elective surgery are diagramed as confounders sharing negative associations with underinsurance but positive associations with higher tidal volume. C. Theorized causal association diagram between minority race/ethnicity (exposure) and higher tidal volume (outcome). Underinsurance, more comorbidities, and female gender are diagramed as potential confounders sharing positive associations with the exposure and outcome.

Our hypothesized causal diagram indicated that height may mediate the association between gender and lung-protective ventilation [24] (Figure 3).

When we adjusted for patient height, the association between gender and tidal volume > 8 mL/kg PBW was substantially weakened (OR = 1.28 [0.91-1.80), demonstrating that height was a strong mediator of the gender and tidal volume relationship. However, to examine whether gender plays a role in tidal volume choice in subgroups of taller and shorter patients, we performed stratified analysis with dichotomous height classification using the median height of 5 feet 7 inches of all patients, as in prior research [17]. We found that gender-based differences in tidal volume > 8 mL/kg PBW occurred both in shorter patients (OR = 1.66, 95% CI = 1.13-2.42) and taller patients (OR = 1.82, 95% CI = 1.14-2.91). This suggests that gender continued to play a role in tidal volume selection despite gender differences in height. Furthermore, the effect estimate for gender was similar across height categories (as above, 1.66 vs 1.82). The lack of heterogeneity between these effect sizes indicates that height is not a significant effect-modifier for the gender-tidal volume relationship, that is, we did not find an interaction between height and gender in predicting tidal volume (see “effect modification (interaction) analysis” in the supplement).

Mediation analysis further explored the relationship between gender and height in predicting tidal volume (see “mediation analysis” section of supplement and Supplementary Tables 8-12). This analysis indicates that a direct effect of female gender on choice of tidal volume was operative in approximately 39% of cases where the provision of tidal volume > 8 mL/kg PBW was related to gender and/or height. Likewise, an indirect mediation pathway, where gender affects height, which, in turn, affects tidal volume choice, was operative in 59% of cases where the provision of tidal volume > 8 mL/kg PBW was related to gender and/or height.

Our hypothesized causal diagram (Figure 3 and Supplementary Table 13) modeled age and comorbidity as variables that could be associated with height (the mediator) and tidal volume choice (the outcome) [25,26]. Multivariable analysis with these covariables demonstrated similar findings to the analysis adjusting for height alone (Table 3). These results indicate minimal influence of age and comorbidity on the gender-height-tidal volume relationship.

Relationship Between Race/Ethnicity and Lung-Protective Ventilation

Unadjusted and PBW-adjusted tidal volumes were similar among racial and ethnic categories (Table 4).

**Table 4:** The relationship of lung-protective ventilation with race/ethnicity Note: Values refer to median (interquartile range) or number (percentage) ‡Adjusted for gender, insurance status, and total number of comorbidities (0-5) Abbreviations: PBW, predicted body weight

	Race					Ethnicity		
	White (n = 1,113)	Black (n = 424)	Asian (n = 51)	American Indian/Alaska native (n = 7)	p-Value	Non-Hispanic or Latino (n = 1,544)	Hispanic or Latino (n = 51)	p-Value
Tidal volume (mL)	450 (400 – 500)	450 (400 – 500)	450 (390 – 500)	350 (300 – 500)	0.15	450 (400 – 500)	450 (400 – 500)	0.61
Tidal volume/PBW (mL/kg)	7.1 (6.2 – 8.0)	7.07 (6.2 – 8.0)	7.6 (6.4 – 8.3)	6.7 (5.9 – 7.3)	0.35	7.1 (6.2 – 8.0)	7.2 (6.4 – 8.2)	0.40
Tidal volume > 8 mL/kg PBW	303 (27)	115 (27)	16 (31)	1 (14)	0.79	419 (27)	16 (31)	0.50
unadjusted odds ratio	1 (reference)	0.99 (0.62 – 1.60)	1.22 (0.61 – 2.44)	0.44 (0.07 – 2.69)		1 (reference)	1.23 (0.49 – 3.08)	
Multivariable adjusted odds ratio (all variables)‡	1 (reference)	0.86 (0.52 – 1.41)	1.30 (0.63 – 2.70)	0.32 (0.05 – 2.00)		1 (reference)	1.08 (0.39 – 2.94)	

These findings were similar after adjustment for gender, insurance status, and comorbidity [27,28] (Table 4; also see Figure 3 illustrating the proposed causal pathway involving these covariables and Supplementary Table 14 detailing relationships between these covariables and race/ethnicity).

Relationship Between Insurance Status and Lung-Protective Ventilation

PBW-adjusted tidal volumes were slightly higher in underinsured compared to insured patients (Table 3). There were slightly more underinsured patients receiving tidal volume > 8 mL/kg IBW when compared to insured patients (31% vs 26%, OR = 1.26, 95% CI = 0.92-1.74).

We considered age, race/ethnicity, comorbidity, and ICU admission after elective surgery as potential confounders of the relationship between insurance status and lung-protective ventilation (Figure 3 and Supplementary Table 15) [27,29]. The association between underinsurance and tidal volume above 8 mL/kg PBW was stronger after adjusting for these covariables (OR = 1.55, 95% CI = 1.15-2.07, Table 3). This masking of the true association is explained by the confounding effects of age and ICU admission after elective surgery. These variables were “negatively” associated with the independent variable of interest (underinsurance) and “positively” associated with the outcome of interest (tidal volume above 8 mL/kg PBW), that is, older patients and the patients admitted to ICU after elective surgery were less likely to be underinsured (negative association) and more likely to receive tidal volume > 8 mL/kg PBW (positive association).

Sensitivity analysis examining the relationship between insurance status and lung-protective ventilation excluding Medicare patients (n = 689) demonstrated similar findings, with multivariable analysis showing that underinsured patients were 71% more likely to receive non-lung-protective ventilation than insured patients (Supplementary Table 16).

Additional post-hoc sensitivity analyses included the addition of severity of illness, presence of ARDS, and mode of mechanical ventilation to the models. None of these variables appreciably affected our results (Supplementary Tables 17-19). We also constructed post-hoc hierarchical models nesting patients within their ICUs. These analyses confirm that our models using ICU clustering accounted for possible differences in care received by patients within individual ICUs (Supplementary Table 20).

Multiple imputation analysis

This analysis combined the patients in the complete case analysis with the 918 patients with one or more missing values for height, tidal volume, or race/ethnicity, yielding 2,513 patients. The imputation model accounted for baseline differences between patients with vs without missing values (Supplementary Table 6). Imputed values were similar to the values recorded in the complete cases (Supplementary Table 7).

The association between gender and tidal volume > 8 mL/kg PBW was similar in magnitude to that observed in the complete case analysis, but now statistically significant in the multivariable logistic regression model including height (OR = 1.37, 95% CI = 1.03-1.83, Supplementary Table 21).

Likewise, the relationship between insurance status and tidal volume above 8 mL/kg PBW was of similar magnitude to that observed in the multivariable analysis of the complete cases (OR = 1.42, 95% CI = 1.06-1.89, Supplementary Table 21). This relationship was similar when exclusively analyzing 1,455 non-Medicare patients in this dataset (OR = 1.48, 95% CI = 1.09-2.02, Supplementary Table 22).

There remained no significant associations between tidal volume above 8 mL/kg PBW and racial/ethnic categories in this larger multiple imputation dataset (Supplementary Table 21).

## Discussion

In this multicenter prospective cohort study of critically ill patients with respiratory failure in the United States, we found that women were less likely to receive lung-protective ventilation compared to men. While height differences between men and women mediate a large portion of this effect, our analysis suggests that gender also has a direct effect on tidal volume choice. Furthermore, we found that underinsured patients were less likely to receive lung-protective ventilation than insured patients after accounting for other imbalances between these groups.

The gender disparity we observed in tidal volume is consistent with Han et al.’ finding that women with sepsis and ARDS are less likely to receive lung-protective ventilation than men [24], a finding attributed to the shorter height of women. A more recent large study including two U.S. ICU cohorts also demonstrated gender differences in tidal volumes, fully explained by the shorter height of women [12]. Our study reinforces these findings in a separate prospective and multicenter cohort of unselected mechanically ventilated critically ill patients.

Height-based differences in care delivery like the one described here could play a role in the inverse relationship that has been observed between height and mortality in the critically ill [30]. These differences may be exacerbated by overestimating height in shorter patients, thus exposing them to excessive tidal volumes [31]. Our dataset did not specify whether heights were measured or estimated. If estimated, our results may be biased toward underestimating the frequency of high tidal volumes in shorter patients, many of whom are women.

Height may be sufficient to explain gender difference in tidal volume [12]. However, our mediation analysis suggests that a direct effect of female gender on tidal volume choice contributed to 39% of the cases in which high tidal volume was related to gender and/or height. In addition, the gender difference in tidal volume was observed in shorter and taller individuals stratified by the median height ≥ 5 feet 7 inches. Finally, our multiple imputation analysis in the larger sample size indicated that gender was associated with tidal volume > 8 mL/kg PBW even after adjusting for height. These three findings suggest the possibility that gender may influence tidal volume choice, even after accounting for height, as shown previously in patients with ARDS [17]. A number of previous studies have reported gender-based disparities in other aspects of ICU care, with less aggressive treatment in women vs men, suggesting gender bias in treatment delivery [3,7,32].

Sex differences in the PBW formula are an additional factor that could contribute to this gender disparity, providing different PBW-based tidal volumes for women vs men of the same height. For example, the 8 mL/kg PBW tidal volume is 493 mL for women 5 feet 7 inches in stature vs 529 mL for men of the same height. If the tidal volume is set at 500 mL for both, only women receive a tidal volume > 8 mL/kg PBW. Although sex-based PBW formulas may be unnecessary for other applications [33], they are appropriate for tidal volume optimization because of sex differences in lung volume [34,35]. Creating ventilator algorithms that calculate and deliver tidal volumes bases on clinician-entered values for sex, measured height, and desired mL/kg PBW tidal volume could more consistently provide lung-protective ventilation than the current practice of ordering absolute unadjusted tidal volume [36].

We found that underinsured patients were less likely than insured patients to receive lung-protective ventilation. To our knowledge, this insurance-based disparity in tidal volume has not been reported previously, though insurance status-based differences in other ICU processes of care are well-known [2,4,27]. Access to acute care probably does not account for this disparity since all patients were receiving critical care at the time of enrollment in our study. Likewise, differences in ICU quality are unlikely to explain our findings since robust variance estimation with ICU-level clustering in our logistic models accounted for the possibility that patients within individual ICUs are correlated. Finally, different treatment preferences or beliefs are unlikely to explain these findings because tidal volume is not a value-sensitive decision and it is improbable that preferences of patient or surrogate decision makers could have influenced tidal volume choice. It is possible that clinicians’ implicit biases influenced their adherence to lung-protective ventilation [37,38], negatively impacting underinsured patients. Prior studies have demonstrated that treatment decisions by clinicians in acute care are influenced by socioeconomic status-based implicit bias [39,40]. Further work is warranted to identify whether insurance-based bias exists in critical care, define its effect on treatment decisions, and test strategies for its elimination.

We did not find racial or ethnic differences in the application of lung-protective ventilation. These results are surprising in the context of numerous studies demonstrating significant racial differences in critical care and outcomes [2,41-43]. Our regression models were clustered by ICU, accounting for potential correlations in processes of care within these ICUs. Prior studies have shown that racial differences in critical care outcomes are attenuated after adjustment for the site (and, by extension, the quality) of care delivery [44,45]. That said, even our unadjusted analyses did not show differences in lung-protective ventilation by race or ethnicity (Table 2).

Our negative findings regarding race and ethnicity may relate to the limitations of our study. Our racial designations were gleaned from the medical records by data abstractors at each site. It is unknown whether these racial designations were consistently recorded in the medical records using the preferred method of self-report [46]. In addition, the medical records frequently contained ambiguous terminology that could not be confidently classified into one of the standard designations [47], contributing to the high number of missing values in our dataset. Even though we did not observe racial/ethnic differences in tidal volume, our analyses demonstrated that minority populations are overrepresented among the underinsured (Supplementary Table 12) and therefore remain at risk of not receiving lung-protective ventilation [27].

Our cohort included all mechanically ventilated patients. Lung-protective ventilation is considered best practice in ARDS, though it is not invariably applied, with average tidal volume of 7.8 mL/kg in ARDS patients across 50 countries [8]. In patients without ARDS, lung-protective ventilation may not be the standard of care, but several studies support its use in these patients as well, showing lower levels of pro-inflammatory cytokines, lower radiographic evidence of lung injury, shorter hospital stays, and less post-operative pulmonary complications [10,11,48-50]. A randomized controlled trial showed no differences in clinical outcomes when patients were randomized to low vs intermediate tidal volume [51], but a large amount of overlap in tidal volume between groups may have biased these results toward the null [52]. Regardless of whether universally accepted in non-ARDS acute respiratory failure, differences in the application of lung-protective ventilation in these demographic groups are an important signal of disparities in ICU care.

Strengths and limitations

Our study has several strengths, particularly the prospective cohort design, manual data abstraction, large sample size, and nationwide ICU representation from 35 medical centers. There are several limitations to our study. First, our observational study design does not permit conclusions about whether there is any causal basis for the associations we observed between tidal volume and gender, height, and insurance status. Likewise, we cannot rule out residual confounding by other unmeasured variables that may explain these associations. Our use of causal models to define potential confounders may be oversimplified and miss important covariables that could be responsible for our findings [19]. For example, we were unable to determine which patients in this dataset had ARDS, and its presence would influence tidal volume. If ARDS were differentially distributed among our demographic groups, this could confound our findings. However, consistent demonstration of disparities in processes of care across different studies increases the likelihood that the similar associations we report are robust [2-4,7,24,39]. Our findings thus add to the evidence suggesting that women, shorter people, and the underinsured are treated differently in U.S. ICUs. Second, our cohort included predominantly academic institutions, and thus its applicability to patients in community hospital may be limited. Third, we collected each patient’s ventilator data only once, and it may have been on any day from 1 to 10 of their ICU stay. This “snapshot” of tidal volume delivery may not accurately reflect the volume received throughout their treatment with invasive mechanical ventilation. Fourth, height, race, and gender were taken from the medical record without specification about how they were originally ascertained. We are unsure whether race and ethnicity were consistently obtained by the recommended method of self-report [46]. If not, there is risk of non-differential ascertainment bias and possible obscuration of true racial differences [53]. Likewise, heights may have been inaccurate if they were estimated instead of measured, with overestimation particularly likely in women, [31] and accompanying risk of differential ascertainment bias. If so, gender differences in lung-protective ventilation may be even larger than we report here. Fifth, it is important to note that the associations we identify in this study may have changed considerably since 2010-2012 when our data was obtained. In this regard, it is important to note that Swart et al. observed an increase in lung-protective ventilation increased between 2001 and 2015, but gender disparities in lung-protective ventilation persisted through this time interval nevertheless [12]. Determining whether the disparities we observed persist in a more contemporary cohort is an important next step.

## Conclusions

This analysis of a large prospective cohort study demonstrates disparities in the provision of lung-protective ventilation in the United States. Women were less likely to receive lung-protective ventilation compared to men, an association largely but not fully explained by the shorter height of women. Furthermore, we find a robust association between underinsurance and non-adherence to lung-protective ventilation especially after accounting for other imbalances between patients with different insurance types. Tidal volume prescription is a clinical management decision. Our findings suggest this decision may be biased by demographic and phenotypic factors such as insurance status, gender, and height. Additional research is required to confirm these findings, evaluate the extent to which implicit bias determines processes of ICU care, and test interventions to eliminate these disparities.

## Appendices

Supplement

Multiple Imputation Methods

Race/ethnicity was missing in 96 patients, height was missing in 147 patients, and tidal volume was missing in 738 patients in the full dataset. STATA/SE 14.2 was used for all multiple imputation analyses. The Stata commands for the imputation step, and the completed data analysis/pooling steps are shown in Supplementary Table 5.

**Table 5:** STATA 14, multiple imputation commands Abbreviations: MI, multiple imputation; APACHE, acute physiology and chronic health evaluation; SOFA, sequential organ failure assessment; HIV, human immunodeficiency virus; ICU, intensive care unit; VCE, variance-covariance matrix estimate

Description	STATA command
Format the data for multiple imputation	MI set wide
Register the variables with missing values that are to be imputed	MI register imputed race height^3^ square root of tidal volume
The imputation model	MI impute chained (regress) height^3^, square root of tidal volume, (logit) race = age, sex, comorbidities, insurance status, admission after elective surgery, APACHE II score, SOFA score, heart failure, cancer, chronic kidney disease, HIV, infection, trauma, admission diagnosis endocrine, admission diagnosis, admission source, ICU type, hospital type nutrition protocol, acute lung injury protocol, daily plan of care, add (40) augment
Fit the model for each imputation and combine the results, gender is primary exposure variable	MI estimate: logistic tidal volume > 8 mL/kg sex age height comorbidities, VCE (cluster site)
Fit the model for each imputation and combine the results, race is primary exposure variable	MI estimate: logistic tidal volume > 8 mL/kg race insurance status comorbidities, VCE (cluster site)
Fit the model for each imputation and combine the results, insurance status is primary exposure variable	MI estimate tidal volume > 8 mL/kg insurance status age post-operative from elective surgery race comorbidities

We assumed that the mechanism for missingness was not dependent on the unobserved data. For example, a patient’s height was not missing because shorter people tended to have height recorded less frequently, a patient’s race was not missing because black patients were less likely to have their race recorded, and a patient’s tidal volume was not missing because patients receiving higher tidal volumes were less likely to have their tidal volumes recorded in the respiratory flowsheets. This was assumed because the variables with missing values are routinely collected and entered into the medical record by hospital staff. Study investigators used this medical record as the primary source of data abstraction. If hospital personnel did not enter a value for one of these fields, it was recorded as missing by study investigators. It seems unlikely that the missing data for these variables depended on the unobserved data, as in the examples above. Instead, it seems more plausible that these data were missing because of human error in the completeness of medical record keeping by hospital staff and that this did not depend on the missing variables themselves. For these reasons, we considered these data missing at random (MAR) [54]. Finally, we did not make any assumptions about the pattern of missingness and instead assumed that missingness was arbitrary [22].

We used multiple imputation using chained equations (MICE) with the “augment” option to avoid perfect prediction as the imputation method [22,55,56]. Our imputation model included the primary outcome variable (tidal volume > 8 mL/kg PBW [predicted body weight]), our pre-specified covariables from the primary multivariable analyses (Figure 3), and all variables predictive of the missing values (those variables differing [p < 0.05] between observations with vs without missing values for race/ethnicity, height, or set tidal volume) [54]. These values included insurance status, APACHE II score, SOFA score, medical history of heart failure, cancer, chronic kidney disease, or HIV infection, a trauma or endocrine admission diagnoses, the source of hospital admission, the ICU type, the hospital type, the presence of nutrition or acute lung injury protocols, or the presence of a daily plan of care (Supplementary Table 6).

**Table 6:** Patient characteristics (all mechanically ventilated patients) and comparison of patients with vs without missing values Note: Values refer to median (interquartile range) or number (percentage). †Mortality status, ICU length of stay, and hospital length of stay were missing in 171 patients. Abbreviations: ht, height; vt, tidal volume; OR, odds ratio; CI, confidence interval; APACHE, acute physiology and chronic health evaluation; SOFA, sequential organ failure assessment; ICU, intensive care unit; COPD, chronic obstructive pulmonary disease; HIV, human immunodeficiency virus; AIDS, acquired immunodeficiency syndrome

Variable	Study population, N = (2,513)	Non-missing ht/vt (n = 1,730)	Missing ht/vt (n = 783)	OR (95% CI)	Non-missing race ethnicity (n = 2,320)	Missing race or ethnicity (n = 193)	OR (95% CI)
Age (years)	61 (50 – 71)	60 (50 – 71)	61 (49 – 72)		61 (50 – 71)	55 (41 – 67)	
Race							
White	1,620 (67)	1,113 (70)	571 (79)	1.60 (1.30 – 1.99)			
Black		424 (26)	137 (19)	0.64 (0.51 – 0.80)			
Asian		51 (3)	13 (2)	0.55 (0.27 – 1.04)			
American Indian/Alaskan native		7 (0.4)	4 (0.6)	1.26 (0.27 – 4.97)			
Ethnicity				0.52 (0.33 – 0.78)			
Non-Hispanic	2,354 (94)	1,602 (93)	752 (96)				
Hispanic	159 (6)	128 (7)	31 (4)				
Gender				0.94 (0.79 – 1.11)			0.73 (0.53 – 1.00)
Men	1,413 (56)	964 (55)	449 (57)		1,291 (56)	122 (63)	
Women	1,100 (43)	766 (44)	334 (43)		1,029 (44)	71 (37)	
Insurance status				0.67 (0.53 – 0.84)			1.99(1.42 – 2.75)
Insured	1,995 (79)	1,340 (77)	655 (84)		1,865 (80)	130 (67)	
Underinsured	518 (21)	390 (23)	128 (16)		455 (20)	63 (33)	
APACHE II score	20 (15 – 25)	21 (16 – 26)	18 (13 – 22)		20 (15 – 25)	18 (14 – 24)	
SOFA score	6 (4 – 9)	7 (4 – 10)	5 (3 – 8)		6 (4 – 9)	6 (4 – 9)	
Hospital mortality^†^				0.76 (0.62 – 0.94)			1.07 (0.76 – 1.50)
No	1,687 (72)	1,136 (70)	551 (76)		1,552 (72)	135 (71)	
Yes	655 (28)	478 (30)	177 (24)		599 (28)	56 (29)	
Hospital length of stay (days)^†^	18 (10 – 30)	17 (10 – 30)	18 (11 – 30)		17 (10 – 30)	19 (12 – 31)	
ICU length of stay (days)^†^	10 (5 – 18)	10 (5 – 18)	11 (5 – 19)		10 (5 – 18)	13 (6 – 20)	
Comorbid conditions							
Heart failure	378 (15)	287 (17)	91 (12)	0.66 (0.51 – 0.86)	353 (15)	25 (13)	0.83 (0.51 – 1.29)
COPD	639 (25)	449 (26)	190 (24)	0.91 (0.75 – 1.12)	594 (26)	45 (23)	0.88 (0.61 – 1.26)
Cancer	557 (22)	351 (20)	206 (26)	1.40 (1.14 – 1.72)	531 (23)	26 (13)	0.52 (0.33 – 0.81)
Chronic kidney disease	375 (15)	284 (16)	91 (12)	0.67 (0.51 – 0.87)	348 (15)	27 (14)	0.92 (0.58 – 1.42)
Chronic liver disease	288 (11)	208 (12)	80 (10)	0.83 (0.62 – 1.10)	257 (11)	31 (16)	1.53 (0.99 – 2.32)
HIV/AIDS	75 (3)	63 (4)	12 (2)	0.41 (0.20 – 0.78)	71 (3)	4 (2)	0.67 (0.18 – 1.82)
Admission diagnosis category							
Respiratory	1,345 (54)	937 (54)	408 (52)	0.92 (0.77 – 1.09)	1,242 (53)	103 (53)	0.99 (0.73 – 1.35)
Infectious	723 (29)	529 (30)	203 (26)	0.81 (0.67 – 0.99)	660 (28)	63 (32)	1.22 (0.88 – 1.68)
Cardiovascular	709 (28)	503 (29)	206 (26)	0.87 (0.72 – 1.06)	663 (28)	46 (23)	0.78 (0.54 – 1.11)
Gastrointestinal	381 (15)	264 (15)	117 (15)	0.98 (0.76 – 1.24)	342 (15)	39 (20)	1.46 (0.98 – 2.13)
Trauma	207 (8)	121 (7)	86 (11)	1.64 (1.21 – 2.21)	180 (8)	27 (13)	1.93 (1.20 – 3.01)
Endocrine	139 (6)	111 (6)	28 (4)	0.54 (0.34 – 0.83)	128 (6)	11 (6)	1.04 (0.49 – 1.96)
Other	383 (15)	257 (15)	126 (16)	1.10 (086 – 1.39)	354 (15)	29 (15)	0.98 (0.63 – 1.49)
Admission source							
Emergency department	1,061 (42)	776 (45)	285 (36)	0.70 (0.59 – 0.84)	983 (42)	78 (40)	0.92 (0.67 – 1.25)
Hospital floor	515 (20)	342 (20)	173 (22)	1.15 (0.93 – 1.42)	475 (20)	40 (21)	1.02 (0.69 – 1.47)
Operating room	406 (16)	267 (15)	139 (18)	1.18 (0.94 – 1.49)	385 (17)	21 (11)	0.61 (0.36 – 0.98)
Outside hospital	415 (16)	281 (16)	134 (17)	1.06 (0.84 – 1.34)	370 (16)	45 (23)	1.60 (1.10 – 2.30)
Other	116 (5)	64 (4)	52 (7)	1.85 (1.24 – 2.74)	107 (5)	9 (5)	1.01 (0.44 – 2.04)
ICU type							
Medical	1,178 (47)	845 (49)	333 (42)	0.78 (0.65 – 0.92)	1,081 (46)	97 (50)	1.16 (0.85 – 1.57)
Surgical	860 (34)	536 (31)	324 (41)	1.57 (1.31 – 1.88)	781 (34)	79 (41)	1.36 (1.00 – 1.86)
Mixed	475 (19)	349 (20)	126 (16)	0.76 (0.60 – 0.95)	458 (20)	17 (9)	0.39 (0.22 – 0.66)
Hospital type							
Private (not-for-profit)	1,637 (65)	1,036 (60)	601 (77)	2.21 (1.82 – 2.69)	1,516 (65)	121 (63)	0.89 (0.65– 1.22,
Private (for profit)	153 (6)	121 (7)	32 (4)	0.57 (0.37 – 0.85)	149 (6)	4 (2)	0.31 (0.08 – 0.82)
Public (non-federal)	708 (28)	562 (32)	146 (19)	0.48 (0.38 – 0.59)	640 (28)	68 (35)	1.43 (1.03 – 1.96)
Federal	15 (1)	11 (0.6)	4 (0.5)	0.80 (0.19 – 2.72)	15 (1)	0 (0)	---
Number of hospital beds	687 (496 – 873)	724 (496 – 885)	615 (470 – 845)		687 (470 – 873)	724 (550 – 800)	
Nutrition protocol	1,515 (60)	980 (57)	535 (68)	1.65 (1.38 – 1.98)	1,210 (52)	112 (59)	1.31 (0.96 – 1.80)
Acute lung injury protocol	2,033 (81)	1,364 (79)	669 (85)	1.57 (1.24 – 2.0)	1,919 (83)	114 (59)	0.30 (0.22 – 0.42)
Daily plan of care	2,138 (85)	1,427 (82)	711 (91)	2.10 (1.59 – 2.79)	1,974 (85)	164 (85)	0.99 (0.65 – 1.55)

We handled variables with skewed distributions by using mathematical transformations to approximate normal distributions prior to the imputation step. Once the imputation step was complete, we back-transformed these variables to their original scale [54].

One or more of height, tidal volume, and race/ethnicity was missing in 833 of the 2,513 patients (33%). We used 40 imputations to exceed this 33% frequency of missing values [55,57]. The values from each imputation were similar to each other and to those from the complete cases, indicating that the imputation model was appropriate and suggesting that the MAR assumption was plausible in the context of this model (Supplementary Table 7) [57].

**Table 7:** Height, tidal volume, and race values from complete cases and each imputation ‡Results expressed as mean (standard deviation). *n = 2,366 for height, n = 1,824 for tidal volume, n = 2,320 for race. †Imputations were performed for all 2,513 patients in the complete dataset

	Height^‡^	Tidal volume^‡^	Race (percent)			
	(inches)	(mL)	White	Black	Asian	American Indian/Alaska native
Actual*	66.5 (4.5)	458 (88)	72	24	3	0.5
Imputation 1^†^	66.6 (4.5)	462 (89)	73	23	3	0.5
Imputation 2	66.6 (45)	462 (89)	73	24	3	0.6
Imputation 3	66.6 (4.5)	461 (89)	73	24	3	0.5
Imputation 4	66.6 (4.5)	460 (88)	73	24	3	0.4
Imputation 5	66.6 (4.5)	461 (89)	74	23	3	0.4
Imputation 6	66.6 (4.5)	459 (88)	73	23	3	0.5
Imputation 7	66.6 (4.5)	460 (89)	73	24	3	0.6
Imputation 8	66.6 (4.5)	461 (87)	73	23	3	0.5
Imputation 9	66.6 (4.5)	461 (89)	73	24	3	0.5
Imputation 10	66.6 (4.5)	461 (88)	72	23	4	0.6
Imputation 11	66.6 (4.5)	459 (87)	73	24	3	0.7
Imputation 12	66.6 (4.5)	461 (89)	73	24	3	0.5
Imputation 13	66.6 (4.5)	460 (88)	73	23	3	0.5
Imputation 14	66.6 (4.5)	460 (89)	73	23	3	0.5
Imputation 15	66.6. (4.5)	459 (88)	74	23	3	0.5
Imputation 16	66.6 (4.5)	461 (88)	73	24	3	0.4
Imputation 17	66.6 (4.5)	460 (88)	73	23	3	0.5
Imputation 18	66.6 (4.5)	461 (88)	73	24	3	0.6
Imputation 19	66.6 (4.5)	460 (88)	73	23	3	0.4
Imputation 20	66.6 (4.5)	459 (88)	73	23	3	0.6
Imputation 21	66.6 (4.5)	459 (87)	73	24	3	0.6
Imputation 22	66.6 (4.5)	459 (87)	73	24	3	0.4
Imputation 23	66.6 (4.5)	461 (89)	73	24	3	0.6
Imputation 24	66.6 (4.5)	461 (90)	73	24	3	0.5
Imputation 25	66.6 (4.5)	459 (88)	73	24	3	0.4
Imputation 26	66.6 (4.5)	461 (88)	74	23	3	0.5
Imputation 27	66.6 (4.5)	459 (88)	73	24	3	0.7
Imputation 28	66.6 (4.5)	459 (89)	73	24	3	0.4
Imputation 29	66.6 (4.5)	460 (89)	73	23	3	0.8
Imputation 30	66.5 (4.5)	461 (87)	74	23	3	0.5
Imputation 31	66.6 (4.5)	460 (88)	73	24	3	0.6
Imputation 32	66.6 (4.5)	459 (88)	73	24	3	0.7
Imputation 33	66.6 (4.5)	461 (88)	74	23	3	0.4
Imputation 34	66.5 (4.5)	460 (88)	73	24	3	0.5
Imputation 35	66.6 (4.5)	458 (87)	73	24	3	0.4
Imputation 36	66.6 (4.5)	460 (89)	73	24	3	0.5
Imputation 37	66.6 (4.5)	460 (88)	73	24	3	0.6
Imputation 38	66.6 (4.5)	460 (90)	73	24	3	0.5
Imputation 39	66.6 (4.5)	462 (88)	73	23	3	0.6
Imputation 40	66.6 (4.5)	459 (88)	73	24	3	0.4

We then completed data analysis using the same covariables and logistic regression method as in the complete case analyses for each imputation set, and the results were pooled. Stability of the resulting effect estimates was assessed by varying the number of imputations between 10 and 40.

Supplementary Results

Effect modification (interaction) analysis: We used the methods of Matthews et al. [58] to investigate the possibility of heterogeneity in the effect of gender on tidal volume > 8 mL/kg PBW by height categories. The following categories were tabulated:

· Proportion of women of lower height receiving tidal volume > 8 mL/kg PBW (265/604): 0.44

· Proportion of men of lower height receiving tidal volume > 8 mL/kg PBW (51/159): 0.32

· The effect of female gender on tidal volume choice (as a proportion) in shorter patients:

o 0.44-0.32 = 0.12

· Proportion of women of higher height receiving tidal volume > 8 mL/kg PBW (23/106): 0.22

· Proportion of men of higher height receiving tidal volume > 8 mL/kg PBW (96/726): 0.13

· The effect of female gender on tidal volume choice (as a proportion) in taller patients:

o 0.22-0.13 = 0.09

· The difference in the effect of female gender on tidal volume choice in shorter vs taller individuals:

o 0.12-0.09 = 0.03

We then calculated the standard error for this difference in the effect of female gender on tidal volume choice in shorter vs taller individuals to be 0.015 [59].

From here, the 95% CI for the difference in the effect of female gender on tidal volume choice in shorter vs taller individuals is 0.00-0.06. Since this CI included zero, we conclude that there is no significant difference in the effect of female gender on tidal volume choice by category of height.

Mediation analysis: Pearl’s mediation formula [23] was employed to assess the extent to which shorter height mediates the effect of female gender on excessive tidal volume. In this analysis, the exposure (X) is female gender, the mediator (Z) is height < 5’7”, and the outcome (Y) is tidal volume > 8 mL/kg PBW. The mediation formula requires calculation of E(Y|x,z): the expected proportion of patients with or without the exposure (X) and with or without the mediator (Z) but with the outcome of interest (Y), given by gx,z, and E(Z|x): the expected proportion of patients with or without the exposure (X) but with the mediator (Z), given by hx. The formulas for calculating these parameters and the calculations themselves are given in Supplementary Tables 8, 9.

**Table 8:** Parameters required for mediation analysis: formulas *x = 0 if gender = male, 1 if gender = female. †z = 0 if height ≥ 5’7”, z = 1 if height < 5’7”. ‡y = 0 if tidal volume ≤ 8 mL/kg PBW, y = 1 if tidal volume > 8 mL/kg PBW. PBW, predicted body weight

Number of observations	Exposure (X)*	Mediator (Z)^†^	Outcome (Y)^‡^	E(Y|x,z) = g_x,z_	E(Z|x) = h_x_
n_1_	0	0	0	n2 / (n1+ n2) = g_0,0_	(n3 + n4) / (n1+ n2 + n3 + n4) = h_0_
n_2_	0	0	1		
n_3_	0	1	0	n4 / (n3+ n4) = g_0,1_	
n_4_	0	1	1		
n_5_	1	0	0	n6 / (n5 + n6) = g_1,0_	(n7+ n8) / (n5+ n6 + n7 + n8) = h_1_
n_6_	1	0	1		
n_7_	1	1	0	n8 (n7+ n8) = g_1,1_	
n_8_	1	1	1		

**Table 9:** Parameters required for mediation analysis: calculations

Number of observations	Exposure (X)	Mediator (Z)	Outcome (Y)	E(Y|x,z) = g_x,z_	E(Z|x) = h_x_
630	0	0	0	g_0,0 _= 0.132	h_0_ = 0.180
96	0	0	1		
108	0	1	0	g_0,1 _= 0.321	
51	0	1	1		
83	1	0	0	_g1,0=0.217_	_h1=0.851_
23	1	0	1		
339	1	1	0	_g1,1=0.439_	
265	1	1	1		

The results are summarized in supplementary Tables 10, 11.

**Table 10:** Percentage of patients with tidal volume > 8 mL/kg PBW depending on whether or not female gender (X) and height < 5’7” (Z) are present (gx,z) PBW, predicted body weight

Female gender X	Height < 5’7” Z	% Getting high V_T_ g_x,z_ = E (Y|x,z)
Yes	Yes	g_1,1_ = 44%
Yes	No	g_1,0_ = 22%
No	Yes	g_0,1_ = 35%
No	No	g_0,0_ = 13%

**Table 11:** Percentage of patients with height < 5’7” (Z) depending on whether or not female gender (X) is present

Female gender X	% with height < 5’7” (Z) h_x_ = E(Z|x)
No	h_0_ = 18%
Yes	h_1_ = 85%

These parameters, in turn, permit calculation of the direct effect of changing X on Y, the indirect effect of changing X on Y via the mediator Z, and the total effect of changing X on Y, accounting for both the direct and indirect pathways. The formulas and results of these calculations are given below:

Direct effect (DE) = (g1,0 - g0,0)(1 - h0) + (g1,1 - g0,1) x h0

DE = (0.22 - 0.13)(1 - 0.18) + (0.44 - 0.32) x 0.18

DE = 0.09 x 0.82 + 0.12 x 0.18

DE = 0.074 + 0.022

DE = 0.096 = 9.6%

Indirect effect (IE) = (h1 - h0)(g0,1 - g0,0)

IE = (0.85 - 0.18)(0.32 - 0.13)

IE = (0.67)(0.19)

IE = 0.127 = 12.7%

Total effect (TE) = [(g1,1 x h1) + g1,0(1 - h1)] - [(g0,1 x h0) + g0,0(1 - h0)]

TE = [(0.44 x 0.85) + 0.22(1 - 0.85)] - [(0.32 x 0.18) + 0.13(1 - 0.18)]

TE = (0.37 + 0.03) - (0.058 + 0.11)

TE = 0.40 - 0.168 = 0.232 = 23%

The analysis and interpretation of these effects are shown in Supplementary Table 12.

**Table 12:** Interpretation of mediation analysis

Effect calculations	Interpretation
1- (IE/TE)=1-(0.127/0.230)= 0.055= 55%	55% of instances of high tidal volume that are related to gender and/or height occur at least in part because female gender is having an effect. This does not exclude concomitant influences of short height on high tidal volume choice in these instances.
1- (DE/TE)=1- (0.096/0.23)= 0.58= 58%	58% of instances of high tidal volume that are related to gender and/or height occur at least in part because short height is having an effect. This does not exclude concomitant direct influences of female gender on high tidal volume choice in these instances.

**Table 13:** Relationship of gender and tidal volume with covariables of interest Abbreviations: PBW, predicted body weight

		Covariables of interest	
	Age	# of comorbidities	Height (cm)
Gender			
Women (n = 710)	62 (52 – 73)	1 (0 – 2)	162 (157 – 167)
Men (n = 885)	60 (49 – 70)	1 (0 – 1)	177 (170 – 182)
p-value	0.01	0.38	<0.001
Tidal volume			
Tidal volume > 8 mL/kg PBW (n = 466)	62 (52 – 74)	1 (0 – 1)	162 (157 – 170)
Tidal volume ≤ 8 mL/kg PBW (n = 1,214)	60 (50 – 71)	1 (0 – 2)	173 (165 – 180)
p-value	0.01	0.002	<0.001

**Table 14:** Relationship of race/ethnicity and tidal volume with covariables of interest Abbreviations: PBW, predicted body weight

	Covariables of interest				
	Gender		Insurance status		Number of comorbidities
	Men (n = 885)	Women n = 710)	Insured (n = 1,257)	Underinsured (n = 338)	
Race					
White (n = 1,113)	640 (72)	473 (67)	953 (76)	160 (47)	1 (0 – 1)
Black (n = 424)	209 (24)	215 (30)	260 (21)	164 (48)	1 (0 – 2)
Asian (n = 51)	33 (4)	18 (2)	40 (3)	11 (3)	0 (0 – 1)
American Indian/Alaska native (n = 7)	3 (0.3)	4 (0..6)	4 (0.3)	3 (0.9)	1 (0 – 2)
p-value	0.01		<0.001		0.01
Ethnicity					
Non-Hispanic or Latino (n = 1,544)	859 (97)	685 (96)	1,224 (97)	320 (95)	1 (0 – 2)
Hispanic or Latino (n = 51)	26 (3)	25 (4)	33 (3)	18 (5)	0 (0 – 1)
p-value	0.51		0.01		0.02
Tidal volume					
Tidal volume ≤ 8 mL/kg PBW (n = 1,160)	738 (83)	422 (59%)	927 (74)	233 (69%)	1 (0 – 2)
Tidal volume > 8 mL/kg PBW (n = 435)	147 (17)	288 (40%)	330 (26)	105 (31%)	1 (0 – 1)
p-value	<0.001		0.08		0.002

**Table 15:** Relationship of insurance status and tidal volume with covariables of interest Abbreviations: PBW, predicted body weight

	Covariables of interest										
	Age	ICU admission after elective surgery			Race				Ethnicity		Comorbidities
	Years	No (n = 1,414)	Yes (n = 181)	White		Black	Asian	American Indian/Alaskan native	Non-Hispanic or Latino (n = 1,544)	Hispanic or Latino (n = 51)	Number
Insurance status											
Underinsured (n = 3,338)	52 (41 – 59)	319 (22)	19 (10)	160 (14)		164 (39)	11 (22)	3 (43)	320 (21)	18 (35)	1 (0 – 1)
Insured (n = 1,257)	64 (54 – 74)	1,095 (77)	162 (90)	953 (86)		260 (61)	40 (78)	4 (57)	1,224 (79)	33 (65)	1 (0 – 2)
p-value	<0.001	<0.001		<0.001					0.01		<0.001
Tidal volume											
> 8 mL/kg PBW (n = 435)	62 (52 – 74)	365 (26)	70 (39)	303 (27)		115 (27)	16 (31)	1 (14)	419 (27)	16 (31)	1 (0 – 1)
≤ 8 mL/kg PBW (n = 1,160)	60 (50 – 71)	1,049 (74)	111 (61)	810 (73)		309 (73)	35 (69)	6 (86)	1,125 (73)	35 (69)	1 (0 – 2)
p-value	0.01	<0.001		0.79					0.50		0.002

**Table 16:** Relationships between insurance status and tidal volume > 8 mL/kg PBW: sensitivity analysis excluding 689 Medicare patients *Values refer to median (interquartile range) or number (percentage). †Values refer to odds ratio (95% confidence interval). ‡Adjusted for age (continuous), ICU admission after elective surgery, race, ethnicity, and total # of APACHE II comorbidities (0-5) Abbreviations: APACHE, acute physiology and chronic health evaluation; PBW, predicted body weight

	Underinsured (n = 338)	Insured (Medicare excluded) (n = 568)
Tidal volume (mL)*	450 (400 – 500)	450 (400 – 500)
Tidal volume/PBW (mL/kg)*	7.1 (6.4 – 8.2)	7.0 (6.2 – 8.0)
Tidal volume > 8 mL/kg PBW (%)	29	27
Unadjusted odds ratio for receiving tidal volume > 8 mL/kg PBW^†^	1.47 (1.04 – 2.09)	1 (ref)
Adjusted odds ratio for receiving tidal volume > 8 mL/kg PBW^†^	1.71 (1.26 – 2.32) ^‡^	1 (ref)

**Table 17:** Relationship between predictors of interest and tidal volume > 8 mL/kg PBW, including adjustment for SOFA score *Adjusted for sex, insurance status, total # of APACHE II comorbidities (0-5), and SOFA score. †Adjusted for age (continuous), ICU admission after elective surgery, race/ethnicity, total # of APACHE II comorbidities (0-5), and SOFA score. ‡ Adjusted for age (continuous), height (continuous), total # of APACHE II comorbidities (0-5), and SOFA score. APACHE, acute physiology and chronic health evaluation; PBW, predicted body weight; SOFA, sequential organ failure assessment

	Insurance status	Gender	Race*			Ethnicity*
	Underinsured vs insured^†^	Women vs men^‡^	Black vs white	Asian vs white	American Indian/Alaskan native vs white	Hispanic vs non-Hispanic
Odds ratio (95% confidence interval)	1.53 (1.14 – 2.04)	1.27 (0.91 – 1.75)	0.86 (0.52 – 1.45)	1.34 (0.64 – 2.77)	0.36 (.0.06 – 2.30)	1.07 (0.37 – 3.03)

**Table 18:** Relationship between predictors of interest and tidal volume > 8 mL/kg PBW, including adjustment for presence or absence of acute lung injury Note: The presence or absence of acute lung injury (now termed ARDS) was determined by site investigators by chart review. This was missing in 26 patients, leaving 1,569 patients. *Adjusted for sex, insurance status, total # of APACHE II comorbidities (0-5), and presence/absence of acute lung injury. †Adjusted for age (continuous), ICU admission after elective surgery, race/ethnicity, # of APACHE II comorbidities (0-5), and presence/absence of acute lung injury. ‡Adjusted for age (continuous), height (continuous), total # of APACHE II comorbidities (0-5), and presence/absence of acute lung injury. APACHE, acute physiology and chronic health evaluation; PBW, predicted body weight

	Insurance status	Gender	Race*			Ethnicity*
	Underinsured vs insured^†^	Women vs men^‡^	Black vs white	Asian vs white	American Indian/Alaskan native vs white	Hispanic vs non-Hispanic
Odds ratio (95% confidence interval)	1.54 (1.14 – 2.07)	1.26 (.090 – 1.76)	0.86 (0.51 – 1.46)	1.36 (0.66 – 2.79)	0.35 (0.05 – 2.40)	0.86 (0.27 – 2.73)

**Table 19:** Relationship between predictors of interest and tidal volume > 8 mL/kg PBW, including adjustment for mode of mechanical ventilation Note: Mechanical ventilation modes were categorized as follows: assist control (n = 845), synchronized intermittent mandatory ventilation (n = 262), pressure support (n = 63), pressure control (n = 6), airway pressure release ventilation (n = 7), high frequency oscillatory ventilation (n = 0), pressure regulated volume control (n = 264), and other (n = 148) *Adjusted for sex, insurance status, total # of APACHE II comorbidities (0-5), and mode of mechanical ventilation. †Adjusted for age (continuous), ICU admission after elective surgery, race/ethnicity, total # of APACHE II comorbidities (0-5), and mode of mechanical ventilation. ‡Adjusted for age (continuous), height (continuous), total # of APACHE II comorbidities (0-5), and mode of mechanical ventilation. APACHE, acute physiology and chronic health evaluation; PBW, predicted body weight

	Insurance status	Gender	Race*			Ethnicity*
	Underinsured vs insured^†^	Women vs men^‡^	Black vs white	Asian vs white	American Indian/Alaskan native vs white	Hispanic vs non-Hispanic
Odds ratio (95% confidence interval)	1.45 (1.08 – 1.95)	1.26 (0.92 – 1.73)	0.83 (0.52 – 1.32)	1.15 (0.54 – 2.44)	0.45 (0.08 – 2.35)	1.00 (0.37 – 2.72)

**Table 20:** Relationship between predictors of interest and tidal volume > 8 mL/kg PBW using hierarchical modeling with patients nested within ICUs Note: hierarchical models were generated using the “xtlogit” command in STATA 14 including ICU as a fixed effect. All four patients in one ICU had the same outcome prediction for tidal volume > 8 mL/kg PBW, so these observations were dropped from the model leaving 1,591 patients for analysis. *Adjusted for sex, insurance status, total # of APACHE II comorbidities (0-5), and mode of mechanical ventilation. †Adjusted for age (continuous), ICU admission after elective surgery, race/ethnicity, total # of APACHE II comorbidities (0-5), and mode of mechanical ventilation. ‡Adjusted for age (continuous), height (continuous), total # of APACHE II comorbidities (0-5), and mode of mechanical ventilation. APACHE, acute physiology and chronic health evaluation; PBW, predicted body weight

	Insurance status	Gender	Race*			Ethnicity*
	Underinsured vs insured^†^	Women vs men^‡^	Black vs white	Asian vs white	American Indian/Alaskan native vs white	Hispanic vs non-Hispanic
Odds ratio (95% confidence interval)	1.47 (1.05 – 2.06)	1.34 (0.94 – 1.92)	0.85 (0.61 – 1.20)	1.09 (0.54 – 2.22)	0.26 (0.19 – 3.67)	1.79 (0.86 – 3.74)

**Table 21:** Adjusted odds ratios for association between exposures of interest and tidal volume > 8 mL/kg PBW, all mechanically ventilated patients (n = 2,513) *Adjusted for sex, insurance status, and total # of APACHE II comorbidities (0-5). †Adjusted for age (continuous), ICU admission after elective surgery, race/ethnicity, and total # of APACHE II comorbidities (0-5). ‡Adjusted for age (continuous), height (continuous), and total # of APACHE II comorbidities (0-5). APACHE, acute physiology and chronic health evaluation; PBW, predicted body weight

	Insurance status	Gender	Race*			Ethnicity*
	Underinsured vs insured^†^	Women vs men^‡^	Black vs white	Asian vs white	American Indian/Alaskan native vs white	Hispanic vs non-Hispanic
Adjusted odds ratio for receiving tidal volume > 8 mL/kg PBW 10 imputations per missing value	1.40 (1.08 – 1.82)	1.39 (1.03 – 1.88)	0.85 (0.56 – 1.30)	1.34 (0.72 – 2.53)	0.33 (0.06 – 1.72)	1.17 (0.61 – 2.27)
Adjusted odds ratio for receiving tidal volume > 8 mL/kg PBW 30 imputations per missing value	1.42 (1.09 – 1.85)	1.38 (1.03 – 1.84)	0.85 (0.55 – 1.32)	1.32 (0.71 – 2.48)	0.32 (0.05 – 2.27)	1.25 (0.68 – 2.30)
Adjusted odds ratio for receiving tidal volume > 8 mL/kg PBW 40 imputations per missing value	1.42 (1.06 – 1.89)	1.37 (1.03 – 1.83)	0.83 (0.53 – 1.29)	1.30 (0.70 – 2.44)	0.31 (0.05 – 2.01)	1.24 (0.67 – 2.30)
Adjusted odds ratio for receiving tidal volume > 8 mL/kg PBW from primary complete case analysis	1.56 (1.16 – 2.10)	1.28 (0.92 – 1.77)	0.86 (0.52 – 1.41)	1.30 (0.63 – 1.41)	0.32 (0.05 – 2.00)	1.08 (0.39 – 2.94)

**Table 22:** Relationships between insurance status and tidal volume > 8 mL/kg PBW in all ventilated patients, multiple imputation analysis excluding 1,058 Medicare patients (n = 1,455) †Adjusted for age (continuous), ICU admission after elective surgery, race/ethnicity, and total # of APACHE II comorbidities (0-5). APACHE, acute physiology and chronic health evaluation; PBW, predicted body weight

	Underinsured vs insured†
Adjusted odds ratio for receiving tidal volume > 8 mL/kg PBW 10 imputations per missing value	1.45 (1.09 – 1.93)
Adjusted odds ratio for receiving tidal volume > 8 mL/kg PBW 30 imputations per missing value	1.49 (1.12 – 1.97)
Adjusted odds ratio for receiving tidal volume > 8 mL/kg PBW 40 imputations per missing value	1.48 (1.09 – 2.02)
